# *eIF4B* mRNA Translation Contributes to Cleavage Dynamics in Early Sea Urchin Embryos

**DOI:** 10.3390/biology11101408

**Published:** 2022-09-27

**Authors:** Florian Pontheaux, Sandrine Boulben, Héloïse Chassé, Agnès Boutet, Fernando Roch, Julia Morales, Patrick Cormier

**Affiliations:** 1 Centre National de la Recherche Scientifique (CNRS), Sorbonne Université, Integrative Biology of Marine Models (LBI2M), Station Biologique de Roscoff, CS 90074, CEDEX, 29680 Roscoff, France; 2 Sorbonne Université, Centre National de la Recherche Scientifique (CNRS), Integrative Biology of Marine Models (LBI2M), Station Biologique de Roscoff, CS 90074, CEDEX, 29680 Roscoff, France

**Keywords:** eukaryotic initiation factor 4B, eIF4B, cell cycle, cleavage dynamics, sea urchin, early embryonic development, protein synthesis, mRNA translation

## Abstract

**Simple Summary:**

Cell division, also known as mitosis, relies on a complex cascade of molecular events that orchestrates the whole process and decides when cells can start dividing. A key factor in this process is protein synthesis, which is carefully regulated inside the cell to assure the timely production of all the proteins required for mitosis. The embryos of sea urchins divide rapidly after fertilization and represent an informative model to analyze the role of protein synthesis regulation during cell cycle progression. For example, the analysis in the 1980s of sea urchin embryos fostered the discovery of Cyclin B, the first representative of a family of proteins that plays a universal role in controlling cell division. This finding was awarded in 2001 with the Nobel Prize in Physiology and Medicine. However, much remains to be learned, and how protein synthesis controls the time and speed of mitosis in a developing embryo is still unclear. For instance, discovering whether the translation of other mRNAs than mitotic cyclins is required to finely regulate the rate of embryonic cleavage has never been tested. In this work, we investigated the role of the translation of an mRNA encoding a protein called eIF4B in the dynamics of embryonic cell division. We showed that newly synthesized eIF4B directly impacts cell division rates in two sea urchin species. Cell divisions are delayed when the production of eIF4B is inhibited in a fertilized egg. Conversely, increased production of eIF4B accelerates mitosis. Therefore, *eIF4B* mRNA translation represents a new means to regulate the pace of embryonic cleavages. Moreover, since eIF4B is a translational regulator, our findings suggest that the function of its mRNA translation is boosting the production of other proteins essential for mitosis. The cells of the sea urchin embryos seem thus equipped with a controlling device capable of modulating cell division rates, a molecular switch that could contribute to coordinating the first steps of development in other animals as well.

**Abstract:**

During the first steps of sea urchin development, fertilization elicits a marked increase in protein synthesis essential for subsequent cell divisions. While the translation of mitotic cyclin mRNAs is crucial, we hypothesized that additional mRNAs must be translated to finely regulate the onset into mitosis. One of the maternal mRNAs recruited onto active polysomes at this stage codes for the initiation factor eIF4B. Here, we show that the sea urchin eIF4B orthologs present the four specific domains essential for eIF4B function and that *Paracentrotus lividus* eIF4B copurifies with eIF4E in a heterologous system. In addition, we investigated the role of *eIF4B* mRNA de novo translation during the two first embryonic divisions of two species, *P. lividus* and *Sphaerechinus granularis*. Our results show that injection of a morpholino directed against *eIF4B* mRNA results in a downregulation of translational activity and delays cell division in these two echinoids. Conversely, injection of an mRNA encoding for *P. lividus* eIF4B stimulates translation and significantly accelerates cleavage rates. Taken together, our findings suggest that *eIF4B* mRNA de novo translation participates in a conserved regulatory loop that contributes to orchestrating protein synthesis and modulates cell division rhythm during early sea urchin development.

## 1. Introduction

The early cleavages of the sea urchin embryo represent a convenient experimental model to dissect the molecular machinery regulating mRNA translation and analyze its specific contribution to cell cycle progression [1,2]. The sea urchin egg, an isolated haploid cell, has completed meiosis and is physiologically blocked in a G1-like cell cycle stage. In these marine organisms, the resumption of mitotic division relies largely on a drastic increase in protein synthesis, as the three first mitotic divisions are independent of mRNA transcription and ribosome biogenesis [3]. Fertilization thus activates a translation regulatory network (TlRN) tightly intertwined with cell division and can orchestrate the recruitment of a large pool of maternally inherited mRNAs [4]. Not surprisingly, this system has attracted the attention of many investigators, and a global picture of the molecular mechanisms controlling translation during sea urchin early development is slowly emerging.

Upon fertilization, the robust activation of cap-dependent translation is primarily due to the release of the eukaryotic Initiation Factor 4E (eIF4E) from its specific repressor, the eIF4E-Binding Protein (4E-BP) [5,6]. The protein eIF4E, a conserved mRNA-cap-binding protein, is then free to interact with eIF4G [7], a large scaffolding protein that binds to eIF3 and eIF4A [8]. Whereas eIF3 is a ribosome-associated translation initiation factor, eIF4A is an RNA-dependent ATPase and RNA helicase that facilitates the scanning of mRNA 5′ UnTranslated Region (UTR) by unwinding its secondary structure [9]. Together, eIF4E, eIF4G, and eIF4A form a complex known as eIF4F, which acts as a molecular intermediary in recruiting capped mRNAs into ribosomes. In eukaryotes, two additional translation factors–eIF4B and its ancient paralog, the eIF4H protein–stimulate eIF4A and eIF4F activity by promoting the coupling of ATP hydrolysis to RNA unwinding [9].

Cyclins and their catalytic kinase subunits CDKs (Cyclin-Dependent Kinases) govern cell cycle progression [10]. Genetic analysis of *Drosophila* demonstrated that three distinct cyclins (B, A, and B3) make overlapping contributions to mitosis [11,12]. The requirement of mitotic cyclin synthesis for the first mitotic division following fertilization was first inferred from the inhibition of total protein synthesis in clams and sea urchins [13]. *Cyclin B* mRNA translation depends on mTOR activity, inducing eIF4E release from its inhibitor 4E-BP following sea urchin egg fertilization [14]. However, an unfertilized egg is endowed with significant maternal cyclin B protein stores that were proposed as serving early cleavage division [15,16]. Furthermore, since the three mitotic cyclins are intended to make overlapping contributions to mitosis, the inhibition of the first mitotic divisions of the sea urchin embryo by emetine cannot be explained simply by blocking the production of cyclin B. Moreover, in addition to the three mitotic cyclins, the translation of mRNAs encoding still-unidentified molecular players could be required for the correct orchestration of embryonic cleavages. We therefore investigated whether the translation of other mRNAs could influence the dynamics of embryonic cleavage.

Using polysome profiling coupled with high-throughput sequencing, we have demonstrated that fertilization triggers the polysomal recruitment of a large set of maternal mRNAs in the sea urchin *Paracentrotus lividus* [4]. Strikingly, among other mRNAs, *eIF4B* is newly recruited into polysomes during the egg-embryo transition in *P. lividus*. Therefore, we hypothesized that *eIF4B* mRNA neotranslation can be involved in the first mitotic divisions induced by fertilization in sea urchins. Its potential involvement is all the more interesting as it is the only mRNA encoding an eIF4 factor recruited into polysomes after fertilization [17]. Thus, *eIF4B* mRNA de novo translation could participate in a hitherto unknown regulatory mechanism controlling the activation of protein synthesis and, consequently, the dynamics of embryonic cleavage.

There is abundant evidence indicating that eIF4B interacts physically with other components of the translation initiation machinery. Indeed, the mammalian eIF4B homologs display four functional domains that bind to some of its core components. These motifs include a conserved RNA recognition motif (RRM), which is known to interact with the 18S rRNA. In addition, this protein harbors a second RNA binding domain called ARM (arginine-rich motif), an N-terminal poly(A)-binding protein (PABP) interacting domain, and a DRYG (aspartic acid, arginine, tyrosine, and glycine) region that mediates homodimerization and interaction with eIF3. Compared with other eIF4 factors, the eIF4B sequence is less conserved across species. It also seems that this protein is not required for ribosome recruitment, suggesting that eIF4B might be part of the regulatory module contributing to enhancing translational efficiency in specific contexts [18,19,20,21]. Besides, it has been proposed that eIF4B and eIF4H can exert overlapping functions, although eIF4H only presents an RRM motif and lacks the other eIF4B typical domains [22].

To investigate the role of *eIF4B* mRNA polysomal recruitment at fertilization, we generated morpholino oligonucleotides (MOs) to impair *eIF4B* mRNA de novo translation in *P. lividus* fertilized eggs. We observed that the injection of an MO directed against *eIF4B* results in a down-regulation of global protein synthesis and provokes a significant delay of the two first mitotic divisions. Conversely, the overexpression of *eIF4B* stimulates mRNA translational activity and accelerates the onset of the two first mitotic divisions. Moreover, experimental manipulation of the *eIF4B* levels elicits identical phenotypes in *Sphaerechinus granularis*, a second sea urchin species separated from *P. lividus* by at least 30 million years of evolution [23,24]. Our results indicate that *eIF4B* mRNA de novo translation represents a new means to reinforce translation after fertilization. Furthermore, our data suggest that eIF4B protein synthesis is an additional process to the well-known cyclin production to control the dynamics of embryonic cleavages.

## 2. Materials and Methods

### 2.1. Chemicals

Sodium orthovanadate (S6508), EDTA (225658), dithiothreitol (DTT-D9779), N-2-hydroxyethylpiperazine-N’2-ethanesulfonic acid (Hepes-H3375), sodium fluoride (7681-49-4), p-nitrophenyl phosphate (N1891), leupeptin (L2023), aprotinin (A4529), Protease inhibitor cocktail (P8849), ATP (A6419), Tween 20 (P1379), puromycin (P8833), RNase A (55674), Acetylcholine chloride (A6625), and CF™ 488A Hydrazide (SCJ4600015) were obtained from Sigma-Aldrich (Saint-Quentin-Fallavier, France). The m^7^GTP-Sepharose beads (AC-151S) were purchased from Jena Bioscience, Jena, Germany. Mouse monoclonal antibody directed against rabbit eIF4E (AB_397664) was purchased from Transduction Laboratories (Lexington, KY, USA). Horseradish peroxidase-coupled secondary antibodies (P0447) were obtained from Dako SA. Pierce ECL Plus substrate (32134) was purchased from ThermoFisher Scientific (Illkirch-Graffenstaden, France).

### 2.2. Sequenced Used

NP_001408.2 (Hsap_eIF4B); XP_002711054.1 (Ocun_eIF4B); XP_025001367.1 (Ggal_eIF4B); XP_041036169.1 (Ccar_eIF4B); GEDS01037963.1 (Pliv_eIF4B); GAVR01006947.1 and GAVR01069715.1 (Sgra_eIF4B); XP_038044277.1 (Pmin_eIF4B); XP_033127317.1 (Ajap_eIF4B); NP_071496.1 (Hsap_eIF4H); XP_008247306.2 (Ocun_eIF4H); NP_001376228.1 (Ggal_eIF4H); XP_041052609.1 (Ccar_eIF4H); GEDS01027092.1 (Pliv_eIF4H); GAVR01080638.1 (Sgra_eIF4H); XP_038056321.1 (Pmin_eIF4H); XP_033115958.1 (Ajap_eIF4H).

### 2.3. mRNA Production

The nucleotide sequence corresponding to the *P. lividus* eIF4B coding region, a copy of TSA GEDS01037963.1, was synthesized in vitro by GeneArt Life Technologies SAS. The Renilla luciferase (R-Luc) sequence was encoded by the pGb-Eg2-410D2-hxG-A65 plasmid [25]. Both ORF sequences were cloned into the pT7TS vector, which carries the Firefly luciferase (F-Luc) coding region flanked by the UTR regions of the *Xenopus laevis* β-Globin [26]. R-Luc and eIF4B ORFs were amplified by PCR with the In-Fusion CloneAmp Hifi PCR Premix (Takara Bio-638500, Saint-Germain-en-Laye, France) and inserted in place of the F-Luc using the In-Fusion HD Cloning Kit (Takara Bio-639648). All primers were synthesized by Eurogentec. The resulting plasmids were then transformed into DH5α Stella competent cells (Takara Bio-636763) and verified by direct sequencing (Eurofins/GATC services). Capped mRNA preparation was performed as previously described [27].

### 2.4. In Vitro Translation Assays

In vitro translation assays were performed with the Retic Lysate IVT Kit (Invitrogen-AM1200, Villebon Sur Yvette, France) following the manufacturer’s instructions and using the *F-Luc* mRNA (12.5 ng/µL) as a reporter. The translation efficiency of this transcript was analyzed in the presence of either *P. lividus eIF4B* or *R-Luc* mRNAs, both at a final concentration of 12.5 ng/µL. Incubations were performed at 30 °C in an Eppendorf ThermoMixer with agitation at 300 rpm. For each time point, 10 µL of the reaction assay was mixed with 50 µL of ONE-Glo reagent (Promega-E6110, Charbonnières-les-Bains, France), and the sample luminescence was measured for 10 s on a 96-well microplate using a Tristar luminometer (Berthold).

### 2.5. In Vitro Biotinylated eIF4B Co-Purification with eIF4E

In vitro translation of *P. lividus eIF4B* (12.5 ng/µL) or *F-Luc* (3.1 ng/µL) mRNAs was performed using the Retic Lysate IVT Kit in the presence of a pre-charged ε-labeled biotinylated lysine-tRNA complex, according to the manufacturer’s instructions (Transcend kit (L5080) from Promega, Charbonnières-les-Bains, France). This experiment used the *F-Luc* mRNA to produce a control protein for nonspecific binding to m^7^GTP-beads. Each translation reaction contained 204 µL of rabbit reticulocyte lysate, 3 µg of Transcend tRNA, and an amino-acid mix (80 mM) for a total volume of 300 µL. After 2 h of incubation at 30 °C in an Eppendorf ThermoMixer, a volume of 2× binding buffer was added (40 mM HEPES pH 7.4, 100 mM sodium fluoride, 10 mM ATP, 20 mM tetrasodium pyrophosphate (PPi), 100 mM NaCl, 0.4 mM EDTA, 2 mM dithiothreitol (DTT), 1 mM AEBSF, protease inhibitor cocktail and 20 µg/mL of aprotinin and leupeptin). Extracts were then incubated for 15 min at 37 °C with 20 µg/mL RNase A. Subsequent isolation of eIF4E and its bound partners was performed using m^7^GTP-Sepharose beads (Jena Science) as previously described [5]. Briefly, extracts were mixed with 25 µL of beads and incubated at 4 °C for 2 h. After three washes with 1 mL of 1× binding buffer (100 mM NaCl), beads were resuspended in 50 µL of 2× loading buffer (NuPAGE 4X buffer (Invitrogen-NP0008) supplemented with 200 mM DTT). Proteins were resolved by SDS-PAGE on a 12% acrylamide Mini-PROTEAN TGX precast protein gel (Bio-Rad-4561046, Marnes-la-Coquette, France) and analyzed by Western blot analysis. Biotinylated *P. lividus* eIF4B protein was detected using streptavidin-HRP (1/10,000) (Promega-L5061). eIF4E was revealed with a specific primary antibody (1/5000) and a horseradish peroxidase-coupled secondary antibody (1/10,000). Upon incubation with the ECL kit, luminescent signals were detected with a Fusion FX camera (Vilber-Lourmat, Marne-la-Vallée, France).

### 2.6. Handling of Gametes and Embryos

*Paracentrotus lividus* and *Sphaerechinus granularis* adult specimens, collected in the Brest area (France), were supplied by the CRBM facility (Centre de Ressources Biologiques Marines of the Roscoff Biological Station). Gamete spawning was induced by intracoelomic injection of 0.1 M acetylcholine, and eggs were raised at 16 °C in 0.22 µm Millipore-Natural filtered seawater (NFSW). Prior to injection, eggs were resuspended in 10 mL of NFSW containing 3.5 mM citric acid (pH 5) and dejellied for 1 min by swirling in an agarose-coated Petri dish (1% agarose in NFSW) as described in [27].

### 2.7. Determination of Cleavage Rates

Dejellied unfertilized eggs were manually aligned on 1% protamine sulfate-coated (Sigma-Aldrich -P4020) dishes and microinjected with specific morpholino antisense oligonucleotides (MOs). The FemtoJet microinjection system (Eppendorf, Hamburg, Germany) resulted in the bolus injection of approximately 1% of the volume of each egg [28]. In all experiments, scramble MO (Mo-CTL) or mismatch MO (MisMo-eIF4B) were injected in parallel into sibling embryos. Phenotypical rescue experiments were performed by coinjecting the mRNA containing eIF4B ORF flanked by the UTR regions of the *Xenopus laevis* β-Globin (see mRNAs production section) and consequently not targeted by the MO. All MOs were furnished by GeneTools (specific sequences are detailed in Supplementary Table S1). Both MOs and mRNA were diluted at the indicated concentrations in RNase-free water containing 1 mM CF™ 488A Hydrazide, an inert fluorescent dye facilitating bolus visualization [29]. One hour after injection, fertilization was triggered by the addition of diluted sperm. Fertilization efficiency was checked by observing the fertilization membrane raised after sperm addition. Cleavage progression was then monitored under an inversed Leica video microscope during the following 5 h. All injected embryos were imaged every 20 min, and cell divisions were detected using the cell counter plugin of Fiji [30].

### 2.8. In Vivo Translation Assays

Unfertilized dejellied eggs were aligned and stuck on dedicated surfaces of protamine sulfate-coated coverslip pieces as described in [27]. *Fluc* mRNA (100 ng/μL) was co-injected with either *eIF4B* mRNA (100 ng/μL) or *R-Luc* mRNA (100 ng/μL). Fertilization was induced one hour following injection. The volume of the solution introduced by microinjection into each egg was standardized by measuring the CF™ 488A Hydrazide signal. For this purpose, the fluorescent signal was quantified from images acquired under the microscope 30 and 90 min after microinjection. Eggs showing a fluorescence decrease higher than 15% between the two acquisitions were considered lethally affected and consequently discarded from the downstream analysis. At the indicated time after fertilization, the injected embryos were collected and lysed in One-Glo luciferase assay buffer (Promega France, Charbonnières-les-Bains, France), and luminescence was measured in a TriStar luminometer (Berthold) as described in [27].

### 2.9. Polysome Gradient Profile and Analysis of the Translational Status of eIF4B mRNA

We collected egg samples from two different *S. granularis* females, both prior to fertilization and at 90 min post-fertilization. We made mRNA polysomal gradients for each sample using standard protocols adapted to the sea urchin [4,27,31]. We performed RNA reverse transcriptions for each fraction of the gradient as described in [4]. Quantitative PCR was assembled in a 5 µL reaction volume with the SybrGreen qPCR Roche kit (LightCycler 480 SYBR Green I Master-4707516001), using a JANUS MINI dispensing automate (Perkin Elmer; KISSF platform, SBR). For each independent biological replica, qPCR reactions were performed in triplicate on a Light Cycler 480 (Roche, Basel, Switzerland), diluting the RT reactions (1/150th). We used two eIF4B specific qPCR primers that produce a unique 150 bp amplicon laying on the open reading frame. Forward [CCCGTAGACACTGCTGCTAA] and Reverse [GTCTCTCCTTACACGGGCAG]) primers were designed by the Primer3 website (http://bioinfo.ut.ee/primer3/, accessed on 12 October 2020) and synthesized by Eurogentec. The eIF4B pair was efficiently approved with favorable amplification parameters with extracted RNA, and showed a linear amplification over a six-log dilution (1/37.5–1/1200th). Using the cycle threshold (CT) values, the value in each gradient fraction was normalized to the value in the first fraction by the 2ΔCT method. Finally, the distribution of the mRNA on the polysome gradient was expressed as the percentage of total mRNA [32].

### 2.10. Statistical Analysis

Statistics were performed using GraphPad Prism v8.0.2 for Windows (GraphPad Software). *t*-test or Mann-Whitney tests were done accordingly to Shapiro-Wilk normality-test results.

## 3. Results

**Figure 1:**
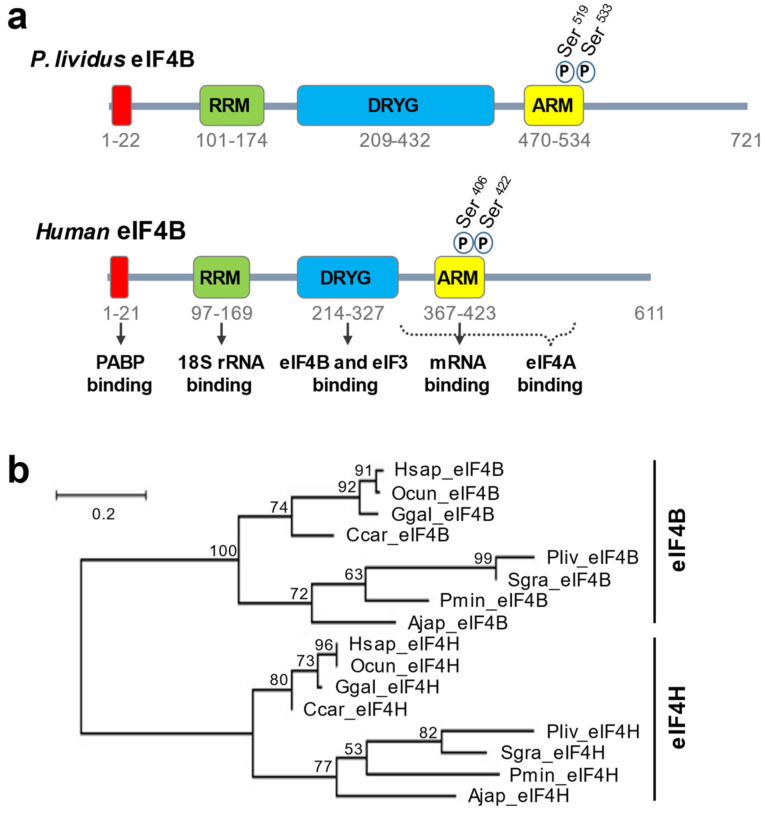
The *P. lividus* eIF4B protein presents four conserved functional domains and is the ortholog of human eIF4B. (**a**) Cartoon depicting the eIF4B protein of *P. lividus* and *H. sapiens*. The four conserved regions with assigned functions in the vertebrate eIF4B appear as colored boxes (RRM, RNA recognition motif; DRYG, aspartic acid, arginine, tyrosine and glycine-rich domain; ARM, arginine-rich motif). The ARM motif harbors two consensus phosphorylation sites also present in the sea urchin homologs. (**b**) Maximum likelihood tree illustrating the phylogenetic relations between eIF4B and its paralog eIF4H. The tree was built with the amino acid sequence of vertebrate and echinoderm RRM domains using a JTT matrix-based model [35], and 500 bootstrap replicates. Branch lengths are proportional to the number of substitutions per site. Bootstrap values are indicated for each branch. RRM domains were found by the software SMART [36]. Hsap: *Homo sapiens*; Pliv: *Paracentrotus lividus*; Sgra: *Sphaerechinus granularis*; Ocun: *Oryctolagus cuniculus*; Ggal: *Gallus gallus*; Ccar: *Carcharodon carcharias*; Ajap: *Anneissia japonica*; Pmin: *Patiria miniata*.

A search for eIF4B homologs in the available genome and transcriptome databases reveals that the echinoderms have a single eIF4B gene. In mammalians, eIF4B presents four specific domains that are essential for its activity [9]. These four domains are found in all sea urchin homologs identified (Figure 1a and Figure S1). Furthermore, we noticed that the RRM 18S rRNA binding motif is remarkably well-conserved in all eIF4B proteins (Figure S1). We thus aligned the sequences of different species to carry out a phylogenetic analysis. This study shows that vertebrate and echinoderm eIF4B sequences cluster together in a single clade, suggesting that these eIF4B homologs form a monophyletic group that separated early in evolution from other RRM containing proteins such as eIF4H (Figure 1b). Moreover, a close comparison of *P. lividus*, *S. granularis,* and human sequences reveals that the vertebrate and echinoderm eIF4B proteins share other important features. For example, although the DRYG region is more extensive in sea urchins than in humans, the main phosphorylation sites of human eIF4B (Ser^406^, Ser^422^) [33,34] are also present in the echinoderm orthologs (Figure 1a and Figure S1). Thus, sea urchins could be an informative system to study the biological functions of eIF4B in a developmental context.

**Figure 2:**
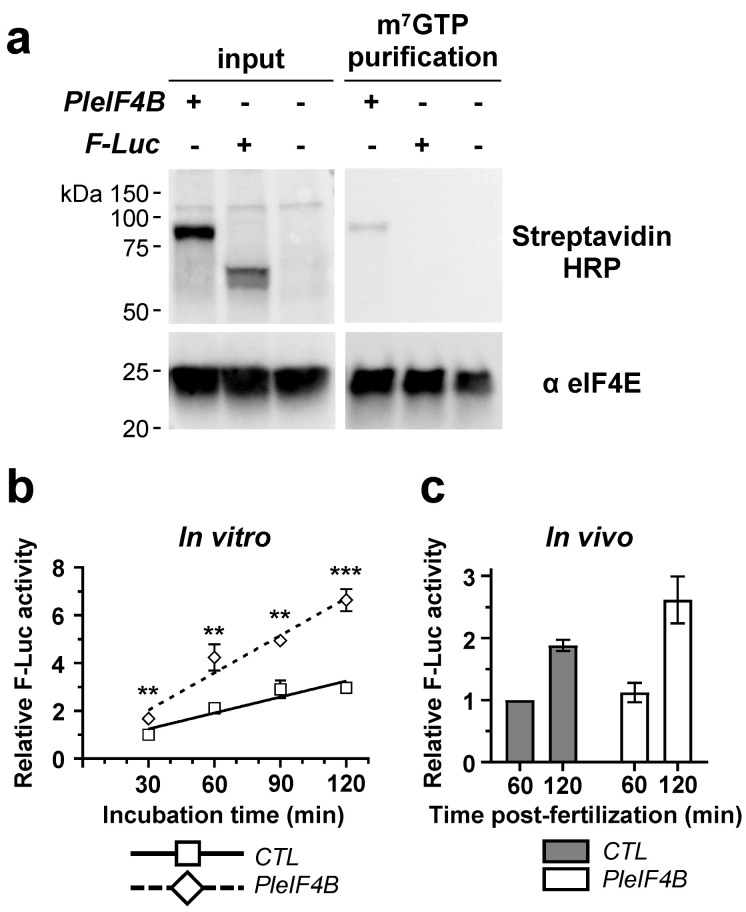
*P. lividus* eIF4B protein copurifies with eIF4E and stimulates translation of a heterologous mRNA reporter. (**a**) Western blot of total protein extracts (left panels) or affinity-purified proteins (right panels) incubated with streptavidin-HRP (top panels) or anti-eIF4E (bottom panels) antibodies. In this experiment, mRNA encoding either for PleIF4B (*PleIF4B*) or F-Luc (*F-Luc*) was added to a rabbit reticulocyte lysate in the presence of biotinylated-lysine-tRNA. Total extracts were treated with RNase and subsequently incubated with m^7^GTP beads. F-Luc protein is used as a control for nonspecific binding to m^7^GTP beads. Input represents 1.6% of the total protein used for affinity purification. (**b**) Plot showing the amount of F-Luc signal measured in rabbit reticulocyte lysates at different incubation times. *Firefly luciferase* mRNA (*F-Luc*; 12.5 ng/μL) was incubated with either *P. lividus eIF4B* mRNA (*PleIF4B*; 12.5 ng/µL) or *Renilla luciferase* mRNA (*CTL*; 12.5 ng/µL). The Firefly luminescence was measured at the indicated times, and values were normalized against those found in the control condition (30 min incubation). Error bars represent SEM for four independent experiments. (**c**) Histogram showing the amount of F-Luc signal found in embryo extracts corresponding to two different developmental stages. *F-Luc* mRNA (100 ng/µL) was coinjected with either *PleIF4B* mRNA (100 ng/µL) or *CTL* mRNA (100 ng/µL). Firefly luciferase luminescence values were normalized against those found in the *CTL* mRNA condition (60 min post-fertilization). Error bars represent SEM for experiments performed in triplicate with the gametes of two different females. Thirty eggs were microinjected for each condition. (Multiple-*t*-test: ** *p*-value < 0.01; *** *p*-value < 0.001).

We aimed to determine whether the sea urchin eIF4B protein can associate with an eIF4F complex. Our attempts to produce a polyclonal antibody against the sea urchin eIF4B protein were unsuccessful, and in our hands, no commercial antibodies against mammalian eIF4B cross-reacted with their echinoderm orthologs. Still, we have demonstrated that sea urchin eIF4B can interact in vitro with an eIF4F complex. For this, a *P. lividus* eIF4B protein showing the expected size (77.4 kDa) was produced in a rabbit reticulocyte lysate. This lysate was then loaded onto a column of m^7^GTP-sepharose beads, which retains eIF4E and its associated proteins (Figure 2a). The purification assay was performed in the presence of RNase A to exclude RNA-mediated interactions. We observe that although eIF4E was effectively purified in all the conditions tested, the beads retained a 77 kDa protein only when the eIF4B lysate was loaded into the column, and not in the control experiments. Thus, the sea urchin eIF4B protein can associate in vitro with a molecular complex containing heterologous eIF4E.

Next, we wondered whether sea urchin *eIF4B* could stimulate mRNA translation rates in an in vitro heterologous system (Figure 2b). For this, translation activity kinetics were monitored in reticulocyte lysates, using *Firefly luciferase* mRNA as a reporter. In these assays, adding an mRNA encoding for a *P. lividus* eIF4B (*PleIF4B*; 12.5 ng/µL final concentration) results in a two-fold increase in luciferase translation rates with respect to controls. Interestingly, this increase is also observed in vivo when we coinject *PleIF4B* and *Firefly luciferase* mRNAs into unfertilized eggs. Indeed, the addition of *PleIF4B* seems to enhance reporter activity, as judged by the luminescent signals obtained at 120 min post-fertilization (Figure 2c). Taken together, these results indicate that eIF4B could contribute to stimulate protein synthesis during sea urchin early development, a role that might be relevant for explaining why its transcript is actively recruited into polysomes after fertilization [4,17].

**Figure 3:**
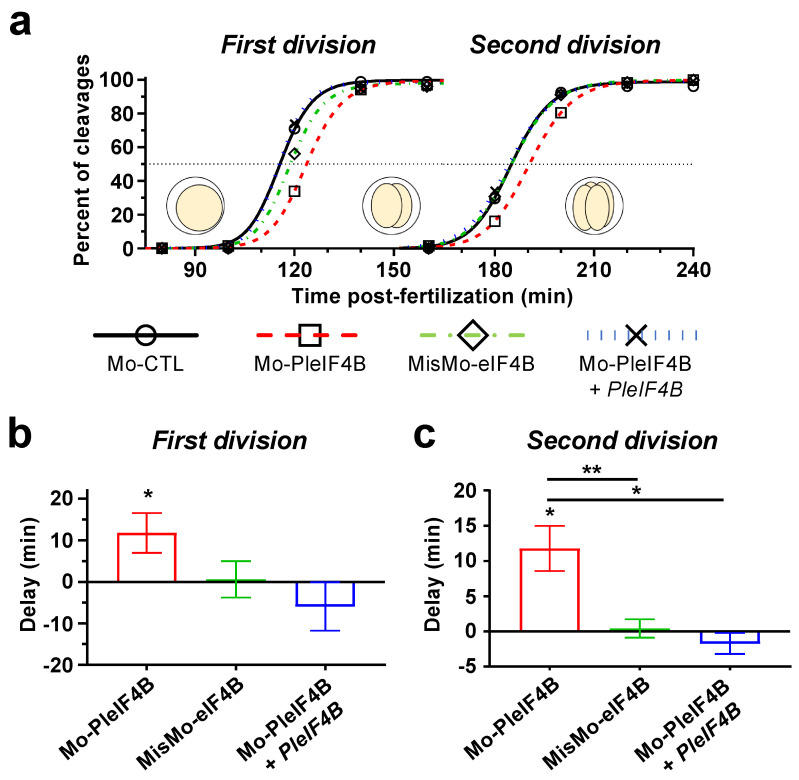
Microinjection of a morpholino directed against *P. lividus eIF4B* mRNA delays the onset of the two first cell divisions. (**a**) Graph showing the cell division kinetics observed in a single representative experiment in which we compare the development of embryos injected with different MOs in the presence or absence of mRNA. The first and second cell divisions are delayed upon injection of a morpholino directed against *P. lividus eIF4B* mRNA (Mo-PleIF4B). As controls, we injected a scrambled morpholino (Mo-CTL), a mismatched morpholino (MisMo-eIF4B), and a rescue *PleIF4B* mRNA, which Mo-PleIF4B does not target. All MOs were injected at 333 µM. Mo-CTL, Mo-PleIF4B, and MisMo-eIF4B were co-injected with 100 ng/µL of *Firefly luciferase* or *PleIF4B* mRNA. This figure is representative of four independent experiments performed with gametes of four different females. (**b**,**c**) Graphs representing the time delay (min) observed at the first (**b**) or second cleavage (**c**) with respect to the values obtained in the Mo-CTL (333 µM) injected embryos used as a reference to calculate delays. Time values correspond to 50% cleavage completion, and positive delays indicate that the embryos develop at a slower pace than the reference population. For each condition, 100 eggs were microinjected, fertilized and monitored throughout early development. Error bars represent the SEM of four independent experiments. (*t*-test: * *p*-value < 0.05; ** *p*-value < 0.01).

We decided thus to determine whether *eIF4B* de novo translation plays a functional role during sea urchin early embryonic development. Accordingly, we designed an antisense morpholino (MO) overlapping with the flanking region of the translation start codon of the *P. lividus* endogenous transcript (Mo-PleIF4B) to block specifically its translation (see Table S1). Then, we injected this MO into *P. lividus* eggs, fertilized them, and monitored the dose-dependent effect of eIF4B knockdown on cell cleavage dynamics (Figure S2). At a concentration as low as 333 µM, Mo-PleIF4B induced an apparent delay in the onset of the two first embryonic cleavages, estimated respectively at 8 min and 12 min. In addition, we confirmed that Mo-PleIF4B provokes a significant decrease in the translation rate, as indicated by the observed activity of a coinjected Firefly luciferase mRNA heterologous reporter (Figure S3). We decided thus to use the same MO concentration for all subsequent experiments. As expected, a morpholino containing five mismatches with the endogenous *P. lividus eIF4B* mRNA (MisMo-eIF4B) did not induce any discernible effect on the dynamics of the two first cleavages, confirming the specificity of the Mo-PleIF4B-phenotypes (Figure 3). Moreover, the Mo-induced cleavage delay phenotype was efficiently rescued by the coinjection of an mRNA encoding eIF4B, which corresponds to the insertion of the PleIF4B coding region flanked by the UTR regions of the *Xenopus laevis* β-Globin, consequently insensitive to Mo-PleIF4B directed inhibition. These data suggest that eIF4B knockdown affects translational rates in vivo and delays the two first cleavages of *P. lividus* embryos.

**Figure 4:**
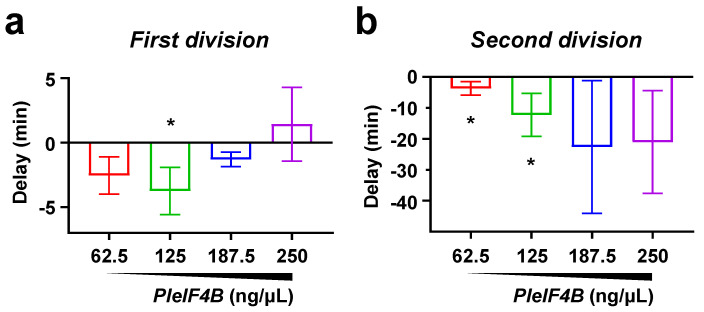
Microinjection of *P. lividus eIF4B* mRNA accelerates cell division. (**a**,**b**) Graphs representing the time delay (min) observed at first (**a**) or second cleavage (**b**) in embryos injected with increasing doses of *PleIF4B*. Times correspond to 50% cleavage completion and values obtained in the 250 ng/µL *Firefly luciferase* mRNA injected embryos were used as a reference to calculate delays. Negative delays thus indicate that the embryos develop faster than the reference population. In each experiment, different amounts of *Firefly luciferase* mRNA were added to reach a constant mRNA concentration of 250 ng/µL. For each condition, 100 eggs were microinjected, fertilized, and monitored throughout early development. Error bars represent the SEM of four independent experiments. (Mann-Whitney test: * *p*-value < 0.05).

We examined whether PleIF4B overexpression could elicit the opposite effect. For this, we injected *PleIF4B* into unfertilized eggs and monitored the dose-dependent effect of this manipulation on the embryo’s first two cleavages (Figure 4). Our results show that, at a concentration of 67.5 or 125 ng/µL, injection of *PleIF4B* mRNA significantly accelerates the rhythm of the two first mitotic cleavages with respect to controls. In contrast, injection of higher amounts of *PleIF4B* mRNA results in division rates comparable to those observed in control embryos, suggesting that an optimal level of eIF4B is required to accelerate development.

**Figure 5:**
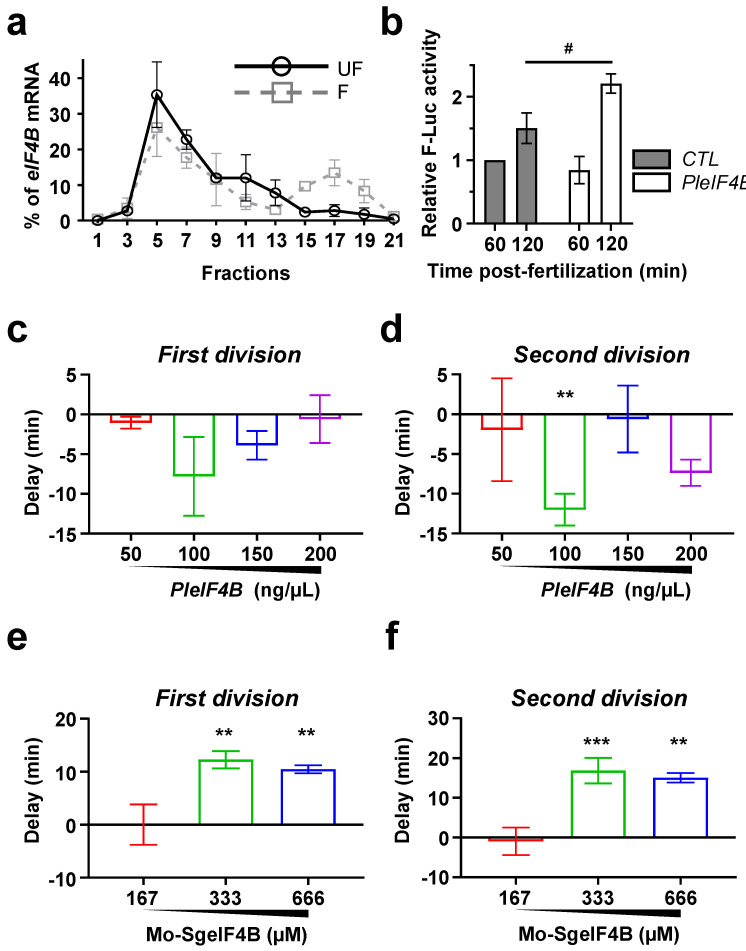
The effects of *eIF4B* mRNA on translation and cell cycle dynamics are conserved in *S. granularis*. (**a**) *eIF4B* mRNA is recruited into polysomes following fertilization. *eIF4B* mRNA was measured by RT-qPCR in each fraction from unfertilized eggs (UF) and 90 min post-fertilization (F). Distribution is shown along the gradient as a percentage of total mRNA. Fraction #1 corresponds to the top of the gradient (free mRNAs), and #21 corresponds to the bottom of the gradient (polysomal fractions). Error bars represent the SEM of two experiments done with independent females. (**b**) *eIF4B* mRNA microinjection stimulates the in vivo mRNA translation activity in *S. granularis* fertilized eggs. *F-Luc* mRNA (100 ng/µL) was introduced by microinjection into the eggs in the presence of either *P. lividus eIF4B* mRNA (100 ng/µL) or *CTL* mRNA (100 ng/µL). Firefly luciferase luminescence was normalized to the *CTL* mRNA condition at 60 min post-fertilization. Error bars represent SEM for experiments performed in triplicate in three independent females. Thirty eggs were microinjected for each condition. (Multiple-*t*-test: # *p*-value < 0.05). (**c**,**d**) Microinjection of *eIF4B* mRNA accelerates the dynamics of the two first cell divisions in *S. granularis* early embryos. Time for fifty percent of first cleavage (**c**) or second cleavage (**d**) was assessed under a light microscope after microinjection of indicated *P. lividus eIF4B* mRNA concentration. In each experiment, constant total mRNA concentration (250 ng/µL) was microinjected by adding the required *Firefly luciferase* mRNA to the microinjection solution. One hundred eggs were microinjected, fertilized and monitored for cell division for each condition. Error bars represent the SEM of three independent overexpression experiments. (**e**,**f**) Microinjection of a morpholino directed against *S. granularis eIF4B* mRNA (Mo-SgeIF4B) delays the dynamics of the two first cell divisions in *S. granularis* early embryos. Time for fifty percent of first cleavage (**e**) or second cleavage (**f**) was assessed under a light microscope. The results are expressed as delay time (in min) compared to the Mo-CTL (666 µM) cell cleavage score. The microinjected solution was prepared with both morpholinos at a total concentration of 666 µM. One hundred eggs were microinjected, fertilized, and monitored for the division for each condition. Error bars represent the SEM of three independent knockdown experiments. (*t*-test: ** *p*-value < 0.01; *** *p*-value < 0.001).

Finally, we sought to establish whether *eIF4B* mRNA de novo translation could play a similar role in *Sphaerechinus granularis*, a sea urchin species belonging to a lineage that separated from *P. lividus* more than 30 million years ago [23,24]. First, we analyzed by qRT-PCR the distribution of *SgeIF4B* mRNA in two polysome gradients prepared with either *S. granularis* unfertilized eggs or 90 min post-fertilization embryos. A comparison of the two distributions reveals that, as in *P. lividus*, *SgeIF4B* mRNA is recruited into active polysomes upon fertilization (Figure 5a and [4,17]). Indeed, in fertilized embryos, *SgeIF4B* mRNA is more abundant in the heavy fractions of the gradient, which accumulate the polysomes (Fractions 15, 17 and 19) (Figure 5a). We could also demonstrate that the injection of *PleIF4B* into *S. granularis* eggs results in a net increase in the F-Luc signal, as observed at 120 min after fertilization (Figure 5b). This indicates that the *F-Luc* mRNA reporter is translated more efficiently in the presence of high levels of eIF4B. In addition, we observed that injection of 100 ng/µL of *PleIF4B* mRNA also accelerates cleavage onset in *S. granularis* embryos (Figure 5c,d).

To further confirm that the role of eIF4B is evolutionarily conserved, we designed a specific morpholino to block endogenous *eIF4B* mRNA translation in *S. granularis* embryos (Table S1). We observed that injection of Mo-SgeIF4B (333 µM) in this species results in a consistent delay of the two first embryonic mitotic divisions (Figure 5e,f). Therefore, *eIF4B* mRNA de novo translation could be part of a conserved mechanism that enhances translational efficiency and contributes to accelerate mitotic division during early development in sea urchins.

## 4. Discussion

The cell cycle has been drastically compressed during the early development of many animal species to accelerate cell divisions and facilitate the rapid hatching of a fully formed embryo. Consequently, the egg’s molecular machinery has acquired different adaptive features that contribute to abridging this development phase in these organisms. In sea urchin embryos, where early development relies to a large extent on the implementation of a translational regulatory network (TlRN) [37,38,39,40], it seems that one of these features is the de novo translation of the *eIF4B* mRNA. Indeed, our results reveal that this initiation factor could play an instrumental role in adjusting translation rates and that its neosynthesis contributes to accelerating cell division rates.

By analyzing the *P. lividus* translatome, we had previously shown that *eIF4B* mRNA is actively recruited into polysomes during the first hour following fertilization, while *eIF4B* mRNA expression levels do not vary [4]. This report shows that *eIF4B* mRNA is also recruited into polysomes in *S. granularis* fertilized eggs, suggesting that *eIF4B*-enhanced translation is a recurrent feature of sea urchin embryos. Furthermore, we provide evidence indicating that this recruitment plays an important biological role, boosting the levels of eIF4B and setting a positive regulatory loop in motion that further enhances the translation of other molecular players.

We have blocked *eIF4B* mRNA translation with specific morpholinos and showed that this provokes a delay in the two first mitotic divisions of *P. lividus* and *S. granularis* embryos, suggesting that *eIF4B* mRNA de novo translation plays a conserved role in controlling cell division rates. This is coherent with previous work demonstrating that eIF4B silencing negatively impacts proliferation in yeast, insect, and mammalian cells [19,20,41,42]. Unfortunately, we do not know which specific cell cycle phase is delayed in the sea urchin embryo, but in these animals, fertilization-induced DNA duplication is independent of de novo translation [43]. It is thus possible that *eIF4B* de novo translation could preferentially impact mitosis entry.

At a mechanistic level, it is known that *eIF4B* mRNA de novo translation is blocked in the presence of mTOR inhibitors, suggesting that mTOR activity is required for *eIF4B* mRNA recruitment into the translational machinery [4,17]. Interestingly, *eIF4B* belongs to a subset of mRNAs specifically regulated by the mTOR complex 1 (mTORC1) in mouse embryonic fibroblasts, where administration of the drug Torin1 suppresses the translation of eIF4B but not the other components of the eIF4F complex [44]. In addition, it is known that LARP1 (La-related protein 1), an RNA binding protein and mTORC1 effector, regulates *eIF4B* mRNA de novo translation in mammals [45]. It would thus be interesting to examine whether a LARP1 homolog contributes to *eIF4B* mRNA translation in sea urchins and if LARP1′s activity impacts mitotic division dynamics.

Although a significant amount of maternally inherited eIF4B protein may already be present in the sea urchin egg, our overexpression studies suggest that eIF4B availability is a limiting factor for mitosis entry. However, an increased dose of *eIF4B* mRNA positively correlates with an accelerated development only if the levels of *eIF4B* do not exceed a certain threshold. This acceleration is not evident when the levels of *eIF4B* are very high. This could be due to a concomitant destabilizing activity exerted by eIF4B upon the molecular complexes that coordinate translation and play an essential role during cell cycle progression [20]. Indeed, it is known that overexpression of eIF4B can provoke disparate effects in mammalian cells, with some studies observing increased translation rates [34,46] and others reporting an inhibitory effect [47,48]. Still, the results of our loss-of-function study indicate that *eIF4B* mRNA de novo translation plays a positive role during mitosis. This is coherent with previous results showing that eIF4B is required in mammals to efficiently translate several mRNAs involved in cell proliferation, such as those coding for Cdc25C, c-Myc, and the ornithine decarboxylase [20]. Moreover, many cell cycle regulators are also translationally activated upon egg fertilization in the sea urchin, including the three mitotic cyclins (A, B, and B3) and CDK1 [4,13,14]. Different mechanisms that underlie the translation control of mitotic player during the cell cycle and development transitions of model organisms have been reported [49]. For instance, eIF4A influences the polysomal association and protein level of the B-type cyclin (Cdc13) in *S. pombe* [50]. Interestingly, the 5′ leader sequence of *Cdc13* presents a conserved feature found in the 5′UTR of *cdc25* mRNA, which is particularly sensitive to limitations of protein synthesis induced by limited eIF4A activity. Therefore, eIF4A could play an essential role in scanning and unwinding the 5′UTR of these mRNAs encoding key mitotic activators in yeast [50]. In sea urchins, the 5′UTR of the mRNAs encoding mitotic players recruited into polysomes after fertilization is currently under investigation. However, since eIF4B stimulates eIF4A and eIF4F activity, the eIF4B protein neotranslated could finely regulate the level of mitotic players required for the fine-tuning of embryonic cleavages.

mTOR pathway inhibition represses *cyclin B* mRNA recruitment partially into active polysome and accumulation of cyclin B protein after fertilization of sea urchin eggs [14]. *eIF4B* mRNA translation is also partially sensitive to mTOR pathway inhibition [17], suggesting a coordinated control of these two molecular players. Therefore, it would be interesting to analyze eIF4B protein production variations in parallel with cyclin B. However, such an approach is conditioned by producing an antibody recognizing sea urchin eIF4B. The partial inhibition of *cyclin B* mRNA recruitment when mTOR is inhibited suggests that an additional mechanism, independent of the 4E-BP level, is involved in its translational regulation. Some reports have put forward 3′UTR-binding proteins in the control of *cyclin B* mRNA translation in different species (review in [49]). For example, in *Xenopus*, CPEB (Cytoplasmic Polyadenylation Element Binding protein) binds a specific sequence in the 3′UTR region of *cyclin B* mRNA, determines polyadenylation status, and consequently, its translational efficiency [51]. *Cyclin B* mRNA translation was reported to be maximal during mitosis and to be driven by the polyadenylation of *cyclin B* mRNA in cycling extracts from *Xenopus* embryos. Sea urchins contain genes involved in cytoplasmic polyadenylation (review in [1]), and egg fertilization is associated with an increase in the polyadenylation of mRNAs [52]. However, sea urchin development before hatching is not affected by cordycepin, an inhibitor of RNA adenylation, suggesting that CPEB-mediated polyadenylation is not required for the first mitotic divisions [53]. Nevertheless, this does not exclude a potential role for the 3′UTR sequence of *cyclin B* mRNA. Indeed, the sea urchin genome contains a PABP that can interact with the poly(A) tail of maternal *cyclin B* mRNA and the eIF4G protein [1]. These simultaneous interactions are likely to form an mRNA closed loop, which is the basis for the observed synergy between the poly(A) tail and the cap in translation [9,54]. The sea urchin eIF4B protein presents potential interaction domains with PABP and eIF3 (Figure 1a and Figure S1). Therefore, PABP interaction with the *cyclin B* mRNA poly(A) tail could promote the association of eIF4B with the eIF4F complex. The synergistic interaction of the neosynthesized eIF4B protein with the 3′ and 5′ UTR regions of *cyclin B* mRNA would thus contribute to controlling its translation. The three cyclins, A, B, and B3, are neotranslated after fertilization and are susceptible to substituting each other. Therefore, it would be necessary to decipher the translation mechanism of these three transcripts to fully understand the potential role of mitotic cyclins’ translational control by eIF4B on embryonic cleavage dynamics.

The mRNA-encoding CDK1 is also newly translated after fertilization [4]. While negligible compared with the amount of maternal protein already present in the egg, the amount of newly synthetized CDK1 associated with cyclin B may have a role in the auto-amplification loop of the mitotic complex [55]. Interestingly, in *Xenopus*, a CDK1-mediated negative feedback loop decreases *cyclin B* translation before mitosis and consequently, improves both the efficiency and robustness of the CDK1-APC oscillator [56]. Therefore, the punctuated cyclin B synthesis drives *Xenopus* early embryonic cell cycle oscillation. In this process, the attenuated cyclin B production involves neither adenylation nor the 3′untranslated region, but is associated with a shift in *cyclin B* mRNA from polysome to non-polysome fractions [56]. Whether newly synthetized CDK1 could regulate cyclin B synthesis in sea urchin embryonic cleavages remains to be tested. Thus, it would be interesting to explore the hypothesis that changes in the cellular levels of eIF4B regulate *CDK1* mRNA translation influencing in fine the pace of division. Eventually, identifying other eIF4B downstream targets is also essential to determining whether eIF4B behaves as a global translational activator or operates more selectively in this specific developmental context.

Our results are consistent with the known molecular role of eIF4B, which contributes in different ways to enhancing mRNA recruitment into the eukaryotic ribosome [57]. In mammalian systems, eIF4B stimulates RNA-dependent eIF4A ATPase activity, ATP-dependent eIF4A RNA binding, and the helicase activity of eIF4A [58,59,60]. As these functions are likely to be conserved, increasing the cellular levels of eIF4B could thus have a direct impact on the activity of the sea urchin embryonic translational machinery. However, other additional regulation events may also be relevant to interpreting the activity of eIF4B in the context of sea urchin early development. For instance, it is known that the phosphorylation of mammalian eIF4B on S^422^ enhances its affinity for eIF3 and that overexpression of the corresponding eIF4B phosphomimetic mutants results in increased translation rates [33,34]. We have shown that the sea urchin eIF4B homologs display several conserved putative phosphorylation sites. Therefore, in these organisms, the global activity of eIF4B may also respond to post-translational modifications altering its phosphorylation status.

## 5. Conclusions

This study reveals that, in addition to the well-known mitotic cyclins, newly synthesized eIF4B contributes to controlling embryonic cleavage dynamics after fertilization in sea urchins. Furthermore, our work provides new insights into the structure of the translational regulatory networks that orchestrate this particular stage of animal life. As eIF4B acts to regulate the translational activity of other mRNAs, our findings suggest that these networks are endowed with positive-feedback loops that can participate in the implementation of a tightly scheduled developmental program and modulate its properties.

A challenge for the future will be identifying the different upstream signaling events that regulate eIF4B neosynthesis and activity, as well as its most relevant molecular targets. This information is essential to clarifing the role of this factor in the context of a translational network controlling cell cycle progression, and could help to understand the logic of inbalances resulting in pathological conditions such as cancer.

## Data Availability

Not applicable.

## Supplementary Materials

The following supporting information can be downloaded at: https://www.mdpi.com/article/10.3390/biology11101408/s1, Figure S1. The sea urchin eIF4B homologs present four conserved domains. Figure S2. Injection of Mo-PleIF4B delays cell division in *P. lividus* early embryos in dose dependent way. Figure S3. Injection of Mo-eIF4B impairs the translation of a heterologous mRNA reporter in sea urchin embryos. Table S1. List of morpholinos used in this study.
