# Supplementary material for: eIF4B mRNA Translation Contributes to Cleavage Dynamics in Early Sea Urchin Embryos
Source: Biology (Basel). 2022 Sep 27;11(10):1408. doi: 10.3390/biology11101408 (PMC9598784; doi:10.3390/biology11101408)
Supplement: Supplementary file 1 [file biology-11-01408-s001.zip › biology-1921168-supplementary.pdf]

Supplementary Materials

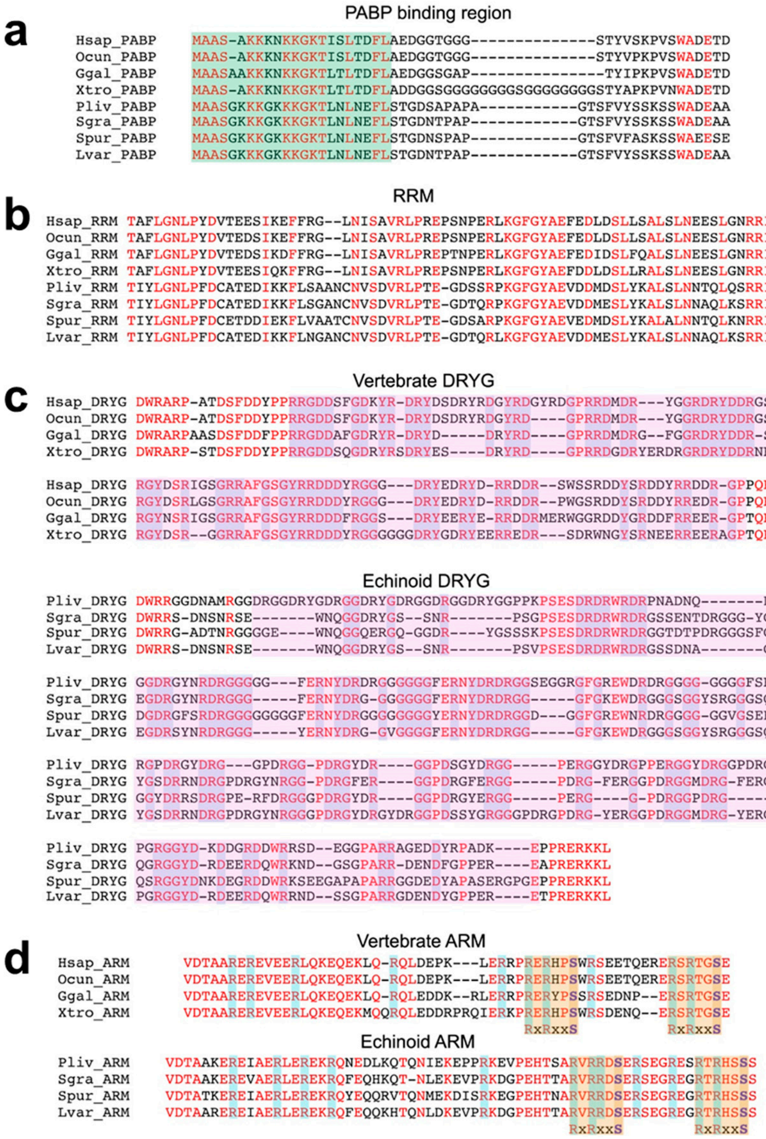

Figure S1. The sea urchin eIF4B homologs present four conserved domains. (**a**,**d**) Clustal-W sequence alignments corresponding to the main functional domains of eIF4B in different vertebrate and echinoid species. Invariant amino acids appear in a red font. (**a**) The N-terminus of eIF4B contains a thoroughly conserved PABP binding region (green box). (**b**) Alignment of the region corresponding to the RRM motif. (**c**) The region containing the DRYG motif (lilac box) is poorly conserved at the sequence level but is rich in D, R, Y and G residues in both clades. These amino acids are marked with a dark hue when fully conserved. (**d**) Region corresponding to the Arginine Rich Motif (ARM). The sequence of this motif differs in the two clades analyzed, but it contains multiple conserved arginine residues marked with blue boxes. Two conserved serine residues (S^{406} and S^{422} in the human sequence) are also marked with a blue font. These serine residues are phosphorylated in the human protein and are part of a consensus Akt kinase recognition motif (RxRxxS, where x represents any amino acid). Consensus motifs are marked with orange boxes. Hsap: *Homo sapiens*; Ocun: *Oryctolagus cuniculus*; Ggal: *Gallus gallus*; Xtro: *Xenopus tropicalis*; Pliv: *Paracentrotus lividus*; Sgra: *Sphaerechinus granularis*; Spur: *Strongylocentrotus purpuratus*; Lvar: *Lytechinus variegatus*. Sequence identifiers: Hsap, NP_001408.2; Ocun, XP_002711054.1; Ggal, XP_025001367.1; Xtro, XP_012812217.1; Pliv, GEDS01037963.1; Sgra, GAVR01006947.1 and GAVR01069715.1; Spur, XP_787362.3; Lvar, XP_041458830.1.

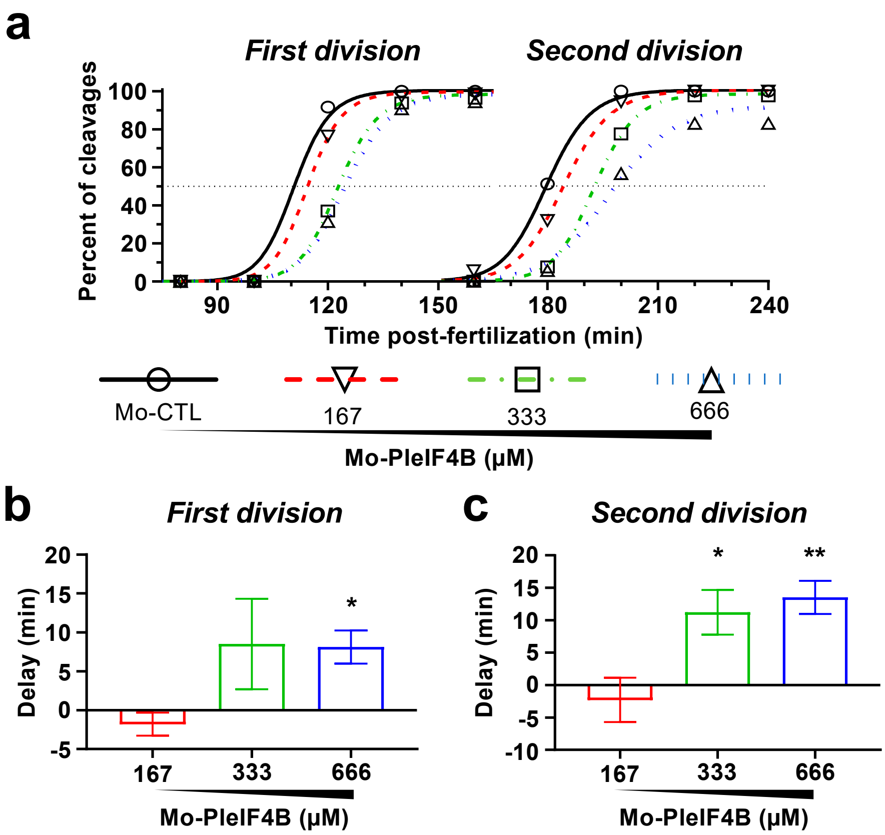

Figure S2. Injection of Mo-PleIF4B delays cell division in *P. lividus* early embryos in a dose-dependent way. (**a**) Graph showing the cell division kinetics observed in a single representative experiment in which we compare the development of embryos injected with different amounts of Mo-PleIF4B. (**b**,**c**) Graphs representing the time delay (min) observed at the first (**b**) or second cleavage (**c**) with respect to the values obtained in the Mo-CTL (666 µM) injected embryos used as reference. In all injection solutions, Mo-CTL was added to reach a constant concentration of 666 µM. Time values correspond to 50% cleavage completion, and positive delays indicate that the embryos develop at a slower pace than the reference population. For each condition, 100 eggs were injected, fertilized and monitored throughout early development. Error bars represent the SEM of three independent experiments. (*t*-test: * *p*-value < 0.05; ** *p*-value < 0.01).

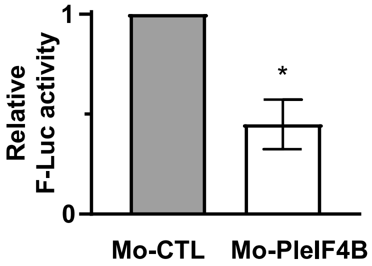

Figure S3. Injection of Mo-eIF4B impairs the translation of a heterologous mRNA reporter in sea urchin embryos. The bars of the histogram show the Firefly Luciferase signal detected in embryos co-injected with *Firefly Luciferase* mRNA (100 ng/µL) and either Mo-PleIF4B or Mo-CTL, both at 333 µM. Firefly luciferase signal was measured 4 h post-fertilization and values were normalized against those found in the Mo-CTL condition. Error bars represent SEM for experiments performed in triplicate using two independent females. In each experiment, thirty eggs were microinjected for each condition. (*t*-test: * *p*-value < 0.05).

Table S1. List of morpholinos used in this study.

Name	Sequence
Mo-CTL	CTCTCCGACAGGTGTTTTGTGACCT
Mo-PleIF4B	CTTACCAGAGGCCGC[CAT]GTTGATA
Mo-SgeIF4B	CTTTCCAGAGGCCGC[CAT]GTTGATA
MisMo-eIF4B [1]	CTTACGACACGCCCC[CAT]CTTGATA

Sequences are given in 5′–3′ direction. Targeted AUG appears in brackets. [1] Introduced changes result in 5 mismatches with the *P. lividus* sequence.
